# The relationship between fibrinogen-to-albumin ratio and brachial-ankle pulse wave velocity in elderly individuals in China: a cross-sectional study

**DOI:** 10.3389/fcvm.2026.1737344

**Published:** 2026-02-09

**Authors:** Yang Zhang, Nan Lu, Yucheng Liu, Jiaxing Ke, Ende Hu, Shanni Chai, Haifeng Chen

**Affiliations:** 1 Department of Cardiology, Shengli Clinical Medical College of Fujian Medical University, Fujian Provincial Hospital, Fuzhou University Affiliated Provincial Hospital, Fuzhou, Fujian, China; 2 Department of Endocrinology, Shengli Clinical Medical College of Fujian Medical University, Fujian Provincial Hospital, Fuzhou University Affiliated Provincial Hospital, Fuzhou, Fujian, China; 3 Department of Intensive Care Unit, First Affiliated Hospital of Fujian Medical University, Fuzhou, Fujian, China; 4 Department of Pediatrics, The Second Affiliated Hospital of Fujian Medical University, Quanzhou, Fujian, China

**Keywords:** arteriosclerosis, brachial-ankle pulse wave velocity, elderly, fibrinogen-to-albumin ratio, vascular aging

## Abstract

**Background:**

Arteriosclerosis, a hallmark of vascular aging, can be assessed using brachial-ankle pulse wave velocity (baPWV). The fibrinogen-to-albumin ratio (FAR), a novel marker reflecting inflammation and hemodynamics, has been proposed as a potential indicator for cardiovascular disease (CVD). However, the association between FAR and baPWV has not been fully elucidated. This study seeks to investigate this association.

**Methods:**

A total of 389 elderly patients were enrolled. Arteriosclerosis was defined as a baPWV ≥1,800 cm/s. Participants were divided into four groups according to FAR quartiles. Multivariate logistic regression was used to assess the association between FAR quartiles and arteriosclerosis. Restricted cubic spline (RCS) analysis was additionally employed to examine the dose–response relationship between continuous FAR and arteriosclerosis risk.

**Results:**

The prevalence of arteriosclerosis increased significantly with increasing FAR quartiles (61.2%, 61.9%, 77.3%, 86.6%; *p* < 0.001). Multivariate linear regression demonstrated an independent positive correlation between FAR and baPWV (*β* = 13.283, 95% CI: 0.286–26.281, *p* = 0.046). In multivariate logistic regression, higher FAR quartiles were linked to higher odds ratios (ORs) for arteriosclerosis (Q2: OR = 0.997, 95% CI: 0.521–1.907, *p* = 0.992; Q3: OR = 2.094, 95% CI: 1.048–4.186, *p* = 0.036; Q4: OR = 2.804, 95% CI: 1.258–6.248, *p* = 0.012) with a significant trend (*p* for trend = 0.002). RCS analysis further confirmed a linear association between FAR and arteriosclerosis risk (*p* for non-linearity >0.05).

**Conclusions:**

In elderly adults, FAR is independently and positively associated with baPWV, suggesting its potential as an additional biomarker for evaluating vascular aging.

## 1 Introduction

The proportion of adults aged ≥65 years worldwide is projected to double from approximately 10% in 2010 to 20% by 2040 (1). With advancing age, the vasculature undergoes structural and functional degeneration, collectively termed “vascular aging,” which has become the leading contributor to disability and mortality in older adults (2). Arteriosclerosis, the hallmark structural manifestation of vascular aging, reflects intrinsic alterations in the arterial wall that increase vascular stiffness and strongly predispose individuals to cardiovascular disease (CVD) (3). In community-dwelling Chinese aged 70–79 years, the rate of prevalence of carotid atherosclerosis has been reported to reach 60%–80% (4). Although carotid-femoral pulse wave velocity (cfPWV) is widely recognized as the gold standard for assessing arterial stiffness, its measurement is time-consuming (5). In contrast, brachial-ankle pulse wave velocity (baPWV) provides a simpler and more efficient alternative (6).

The fibrinogen-to-albumin ratio (FAR) is calculated as the ratio of fibrinogen to albumin in peripheral blood. Fibrinogen and albumin are circulating proteins that exert distinct physiological functions. Fibrinogen is an indicator of a procoagulant state and a biomarker for chronic inflammation (7, 8). Elevated plasma fibrinogen levels are closely associated with an increased risk of major adverse cardiovascular events (MACEs) (9, 10). In contrast, albumin possesses anti-inflammatory, antioxidant, and anticoagulant properties (10–13). Hypoalbuminemia is associated with an elevated risk of MACEs (14, 15). Recent studies have demonstrated that the FAR, a readily available and cost-effective marker, may predict MACEs more accurately than fibrinogen or albumin alone (16, 17). However, the association between FAR and arteriosclerosis has not yet been clearly elucidated.

The present study was designed to investigate the association between FAR and baPWV and to clarify the role of the FAR in evaluating cardiovascular aging.

## 2 Methods

### 2.1 Study patients

This retrospective study was conducted at Fujian Provincial Hospital between 1 January 2018 and 30 June 2019 and included elderly Chinese inpatients admitted during this period. For inclusion in the study, patients had to meet two criteria, the first being aged 65 years or older and the second being having valid brachial-ankle pulse wave velocity (baPWV) measurements obtained using an arteriosclerosis detector. Patients were excluded if they had acute infections, malignancies, acute myocardial infarction, atrial fibrillation, valvular heart disease, myocarditis, aortopathies, acute cerebral infarction, if their ankle-brachial index (ABI) was less than 0.9, if they had used anticoagulants or antiplatelet agents within the previous week, or if they had received a recent blood transfusion. This study was approved by the Ethics Committee of Fujian Provincial Hospital (approval number K2020-12-024). Given that this was a retrospective study, informed consent was waived. A total of 389 eligible participants were ultimately enrolled. Data on participants' medical history [hypertension (HTN), diabetes mellitus (DM)], current medications, smoking status, and other relevant clinical variables were extracted from the hospital's electronic medical records system.

### 2.2 Cardiovascular risk factors

HTN was defined as self-reported hypertension, a blood pressure of ≥140/90 mmHg, or a history of antihypertensive medication use. DM was defined as self-reported diabetes or the use of hypoglycemic agents. Dyslipidemia was defined as triglyceride (TG) ≥1.7 mmol/L, total cholesterol (TC) ≥5.2 mmol/L, low-density lipoprotein-cholesterol (LDL-C) ≥3.3 mmol/L, high-density lipoprotein-cholesterol (HDL-C) <1.0 mmol/L, or the use of antidyslipidemic medications. High body mass index (BMI) was defined as BMI ≥24.0 kg/m^2^. Smoking status was categorized as non-smokers (never smoked) and smokers (former or current smokers).

### 2.3 Physical examination

Clinical assessments encompassed measurements of height, weight, systolic blood pressure (SBP), diastolic blood pressure (DBP), and heart rate (HR). BMI was calculated as weight in kilograms divided by height in meters squared (kg/m^2^). Mean arterial blood pressure (MABP) was calculated using the following formula: MABP (mmHg) = (1/3 × SBP) + (2/3 × DBP).

### 2.4 Biochemical assessment

Blood samples were collected following an 8-h fasting period. The biochemical parameters assessed on admission were platelet count (PLT), albumin (ALB), alanine aminotransferase (ALT), aspartate aminotransferase (AST), fasting blood glucose (FBG), total cholesterol (TC), triglycerides (TG), high-density lipoprotein-cholesterol (HDL-C), low-density lipoprotein-cholesterol (LDL-C), creatinine, uric acid (UA), and fibrinogen. The FAR was calculated as (fibrinogen/albumin) × 100. The estimated glomerular filtration rate (eGFR) was calculated using the CKD-EPI formula, with separate calculations for males and females (18).

### 2.5 Brachial-ankle pulse wave velocity

After 10 min of supine rest, baPWV was measured using a fully automatic arteriosclerosis detector (Colin VP1000; Colin Medical Technology, Komaki, Japan). Bilateral baPWV measurements were acquired, and the higher of the two values was used for analysis. Arterial stiffness was classified into three categories: normal (baPWV <1,400 cm/s), borderline (1,400 ≤ baPWV <1,800 cm/s), and arteriosclerosis (baPWV ≥1,800 cm/s, indicating elevated stiffness) (19).

### 2.6 Statistical analysis

Cardiovascular risk factors, current treatments, and other clinical characteristics were compared between patients with and without arteriosclerosis and across FAR quartile groups. Pearson linear correlation analysis was used to explore potential correlations between FAR and baPWV. Multiple linear regression analysis was performed to quantify the independent association between FAR (continuous variable) and baPWV (continuous outcome). Three adjusted models were constructed sequentially: Model 1 adjusted for gender, age, smoking status, diabetes, hypertension, high BMI, and dyslipidemia; Model 2 further adjusted for current medications (hypoglycemic, antihypertensive, and antidyslipidemic agents); Model 3 additionally adjusted for laboratory covariates (PLT, ALT, eGFR, and UA). The regression coefficient (β) with 95% confidence interval (CI) was reported to reflect the average change in baPWV per unit increase in FAR. Similarly, using the same adjusted models described above, logistic regression analysis was used to evaluate the relationship between FAR and arteriosclerosis. The FAR was treated as either a quartile categorical variable or a continuous variable, while arteriosclerosis was defined as the dichotomous outcome. Restricted cubic spline (RCS) analysis was performed to further characterize the dose–response relationship between FAR and the risk of arteriosclerosis. Three knots (10th, 50th, and 90th percentiles of FAR) were used to balance model complexity and ease of interpretation. Subgroup analyses were performed according to gender (female/male), smoking status (non-smoker/smoker), diabetes (no/yes), hypertension (no/yes), dyslipidemia (no/yes), and high BMI (no/yes). Interaction was tested by adding FAR-by-subgroup product terms to the regression models. All statistical tests were two-sided, and a *p-*value <0.05 was considered statistically significant. Statistical analyses were performed using IBM SPSS Statistics 25.0 software (IBM Corp., Armonk, NY, USA). The RCS analysis was performed using R software version 4.4.0.

## 3 Results

### 3.1 Baseline characteristics

Baseline characteristics of the 389 enrolled patients, stratified by the presence or absence of arteriosclerosis, are summarized in Supplementary Table S1. The cohort had a mean age of 76.5 ± 7.4 years, with 247 (63.5%) males and 101 (26.0%) being smokers. In addition, 167 (42.9%) patients had type 2 diabetes mellitus (DM), 308 (79.2%) had HTN, 261 (67.1%) had dyslipidemia, and 186 (47.8%) had high BMI. The mean FAR was 9.6% ± 4.0%, and the mean baPWV was 2,117.3 ± 531.6 cm/s. Overall, 279 patients (71.7%) had arteriosclerosis. Patients with arteriosclerosis were older and had a higher prevalence of DM and HTN.

Characteristics stratified by FAR quartiles are presented in Table 1. The mean FAR values for each quartile were Q1 (lowest): 6.2% ± 0.8%, Q2: 7.8% ± 0.4%, Q3: 9.5% ± 0.6%, and Q4 (highest): 14.8% ± 4.5%. Fibrinogen levels increased significantly with increasing FAR (*p* < 0.001), while albumin levels decreased significantly (*p* < 0.001). Significant interquartile differences were noted for HDL-C, PLT, UA, and the prevalence of high BMI. No significant differences were observed with regard to age, gender, smoking status, current medication use, ALT, FBG, LDL-C, TG, TC, or the prevalence of DM, HTN, and dyslipidemia.

**Table 1:** Characteristics of patients stratified by quartiles of FAR.

Characteristics	Quartile of FAR				*p-*Value
	Q1 (*n* = 98)	Q2 (*n* = 97)	Q3 (*n* = 97)	Q4 (*n* = 97)	
Cardiovascular risk factor					
Age (year)	76.3 ± 6.7	75.8 ± 8.2	76.9 ± 7.8	76.9 ± 6.8	0.675
Male (*n*, %)	71 (72.4)	56 (57.7)	57 (58.8)	63 (64.9)	0.121
Smokers (*n*, %)	24 (24.5)	22 (22.7)	28 (28.9)	27 (27.8)	0.739
Diabetes (*n*, %)	42 (42.9)	36 (37.1)	43 (44.3)	46 (47.4)	0.529
Hypertension (*n*, %)	78 (79.6)	77 (79.4)	77 (79.4)	76 (78.4)	0.997
Dyslipidemia (*n*, %)	57 (58.2)	65 (67.0)	69 (71.1)	70 (72.2)	0.146
High BMI (*n*, %)	59 (60.2)	46 (47.4)	41 (42.3)	40 (41.2)	0.030
Current treatments (*n*, %)					
Hypoglycemic agents	28 (28.6)	25 (25.8)	34 (35.1)	35 (36.1)	0.337
Antihypertensive agents	62 (63.3)	53 (54.6)	60 (61.9)	47 (48.5)	0.132
Antidyslipidemic agents	19 (19.4)	11 (11.3)	20 (20.6)	13 (13.4)	0.224
Physical exam					
SBP (mmHg)	142.7 ± 18.8	141.5 ± 21.4	143.8 ± 21.2	141.3 ± 22.7	0.828
DBP (mmHg)	77.9 ± 10.8	77.8 ± 11.8	76.2 ± 11.7	74.9 ± 11.2	0.200
MABP (mmHg)	99.5 ± 11.7	99.0 ± 12.9	98.7 ± 12.8	97.0 ± 13.4	0.542
HR (bpm)	75.1 ± 10.2	78.6 ± 12.3	75.4 ± 12.0	77.9 ± 10.3	0.064
Laboratory Data					
PLT (10^9^/L)	201.5 ± 80.1	223.1 ± 57.4	219.5 ± 56.9	273.5 ± 104.4	<0.001
ALT (U/L)	21.5 ± 10.8	20.3 ± 11.9	21.0 ± 12.9	18.0 ± 11.7	0.182
AST (U/L)	22.5 ± 11.4	19.8 ± 6.7	21.2 ± 7.9	19.6 ± 10.5	0.105
FBG (mmol/L)	6.3 ± 2.0	7.1 ± 4.8	6.6 ± 2.6	7.4 ± 3.9	0.128
TG (mmol/L)	1.5 ± 0.8	1.4 ± 0.8	1.5 ± 0.8	1.3 ± 0.9	0.487
TC (mmol/L)	4.4 ± 1.3	4.5 ± 1.1	4.5 ± 1.1	4.3 ± 1.3	0.631
HDL-C (mmol/L)	1.2 ± 0.4	1.2 ± 0.3	1.1 ± 0.3	1.0 ± 0.3	<0.001
LDL-C (mmol/L)	2.8 ± 1.1	2.9 ± 1.0	3.0 ± 1.0	2.9 ± 1.2	0.706
UA (μmol/L)	358.0 ± 102.6	338.4 ± 91.8	351.7 ± 85.7	316.0 ± 107.1	0.015
eGFR (ml/min/1.73 m^2^)	56.8 ± 15.3	58.7 ± 19.5	54.8 ± 17.6	53.3 ± 21.5	0.196
Fibrinogen (g/L)	2.7 ± 0.4	3.3 ± 0.3	3.9 ± 0.4	5.4 ± 1.3	<0.001
ALB (g/L)	43.6 ± 3.6	42.2 ± 3.8	40.9 ± 3.1	36.9 ± 4.2	<0.001
baPWV (cm/s)	1,939.6 ± 416.0	2,083.9 ± 598.8	2,173.0 ± 530.2	2,274.3 ± 516.1	<0.001
Arteriosclerosis (*n*, %)	60 (61.2)	60 (61.9)	75 (77.3)	84 (86.6)	<0.001

BMI, body mass index; SBP, systolic blood pressure; DBP, diastolic blood pressure; MABP, mean arterial blood pressure; HR, heart rate; PLT, platelet count; ALT, alanine aminotransferase; AST, aspartate aminotransferase; FBG, fasting blood glucose; TG, triglyceride; TC, total cholesterol; HDL-C, high-density lipoprotein-cholesterol; LDL-C, low-density lipoprotein-cholesterol; UA, uric acid; eGFR, estimated glomerular filtration rate; ALB, albumin; FAR, fibrinogen to albumin ratio; baPWV, brachial-ankle pulse wave velocity.

### 3.2 Association between FAR and baPWV

BaPWV differed significantly across FAR quartiles, with values increasing progressively with higher FAR levels (*p* < 0.001) (Table 1). The prevalence rate of arteriosclerosis also increased gradually from the lowest to the highest FAR quartiles (61.2%, 61.9%, 77.3%, and 86.6%, *p* < 0.001), with a sharp increase observed between Q2 and Q3 (Table 1).

The Pearson linear correlation analysis showed a positive correlation between FAR and baPWV (*r* = 0.219, *p* < 0.001) (Supplementary Table S2). In linear regression analyses, FAR was positively associated with baPWV (Table 2). In the unadjusted model, FAR was significantly associated with higher baPWV (*β* = 29.189, 95% CI: 16.223–42.156, *p* < 0.001). After adjustment for traditional cardiovascular risk factors (Model 1), the association remained significant (*β* = 23.316, 95% CI: 11.156–35.476, *p* < 0.001). Further adjustment for current medications (Model 2) did not materially change the estimate (*β* = 21.603, 95% CI: 9.293–33.914, *p* < 0.001). After additional adjustment for laboratory covariates (PLT, ALT, eGFR, and UA) in Model 3, the association remained statistically significant (*β* = 13.283, 95% CI: 0.286–26.281, *p* = 0.046).

**Table 2:** Linear regression of FAR and baPWV.

Model	*β* (95% CI)	*p*-Value
Unadjusted	29.189 (16.223–42.156)	<0.001
Model 1	23.316 (11.156–35.476)	<0.001
Model 2	21.603 (9.293–33.914)	<0.001
Model 3	13.283 (0.286–26.281)	0.046

FAR, fibrinogen to albumin ratio; baPWV, brachial-ankle pulse wave velocity; PLT, platelet count; ALT, alanine aminotransferase; UA, uric acid; eGFR, estimated glomerular filtration rate.

Model 1: Adjusted for gender, age, smoking status, diabetes, hypertension, high BMI, and dyslipidemia.

Model 2: Adjusted for gender, age, smoking status, diabetes, hypertension, high BMI, dyslipidemia, hypoglycemic agents, antihypertensive agents, and antidyslipidemic agents.

Model 3: Adjusted for gender, age, smoking status, hypertension, diabetes, high BMI, dyslipidemia, hypoglycemic agents, antihypertensive agents, antidyslipidemic agents, PLT, ALT, eGFR, and UA.

The logistic regression analysis was performed to investigate the relationship between FAR and arteriosclerosis (Table 3). In the unadjusted model, compared with the lowest FAR quartile (Q1), the odds of arteriosclerosis increased across higher quartiles (Q2: OR = 1.027 95% CI: 0.577–1.829, *p* = 0.928; Q3: OR = 2.159, 95% CI: 1.155–4.035, *p* = 0.016; Q4: OR = 4.092, 95% CI: 2.009–8.337, *p* < 0.001; *p* for trend <0.001). When the FAR was modeled as a continuous variable, each 1% increase in the FAR was associated with higher odds of arteriosclerosis (OR = 1.185, 95% CI: 1.088–1.291, *p* < 0.001). These associations remained after multivariable adjustment. In the fully adjusted Model 3, the association persisted (continuous FAR: OR = 1.140, 95% CI 1.037–1.253, *p* = 0.007), and higher FAR quartiles were associated with greater odds of arteriosclerosis (Q2: OR = 0.997, 95% CI: 0.521–1.907; *p* = 0.992; Q3: OR = 2.094, 95% CI: 1.048–4.186; *p* = 0.036; Q4: OR = 2.804, 95% CI: 1.258–6.248, *p* = 0.012; *p* for trend = 0.002).

**Table 3:** Logistic regression of FAR (quartile/continuous) and arteriosclerosis.

FAR	Unadjusted		Model 1		Model 2		Model 3	
	OR (95% CI)	*p-*Value	OR (95% CI)	*p-*Value	OR (95% CI)	*p-*Value	OR (95% CI)	*p-*Value
Q1	1 (ref)		1 (ref)		1 (ref)		1 (ref)	
Q2	1.027 (0.577–1.829)	0.928	1.097 (0.590–2.042)	0.770	1.050 (0.562–1.963)	0.878	0.997 (0.521–1.907)	0.992
Q3	2.159 (1.155–4.035)	0.016	2.015 (1.089–4.227)	0.027	2.211 (1.112–4.395)	0.024	2.094 (1.048–4.186)	0.036
Q4	4.092 (2.009–8.337)	<0.001	4.148 (1.951–8.819)	<0.001	4.063 (1.896–8.704)	<0.001	2.804 (1.258–6.248)	0.012
*p* for trend		<0.001		<0.001		<0.001		0.002
FAR (per 1%)	1.185 (1.088–1.291)	<0.001	1.182 (1.081–1.293)	<0.001	1.182 (1.078–1.295)	<0.001	1.140 (1.037–1.253)	0.007

FAR, fibrinogen to albumin ratio; PLT, platelet count; ALT, alanine aminotransferase; UA, uric acid; eGFR, estimated glomerular filtration rate.

Model 1: Adjusted for gender, age, smoking status, diabetes, hypertension, high BMI, and dyslipidemia.

Model 2: Adjusted for gender, age, smoking status, diabetes, hypertension, high BMI, dyslipidemia, hypoglycemic agents, antihypertensive agents, and antidyslipidemic agents.

Model 3: Adjusted for gender, age, smoking status, hypertension, diabetes, high BMI, dyslipidemia, hypoglycemic agents, antihypertensive agents, antidyslipidemic agents, PLT, ALT, eGFR, and UA.

**Figure 1:**
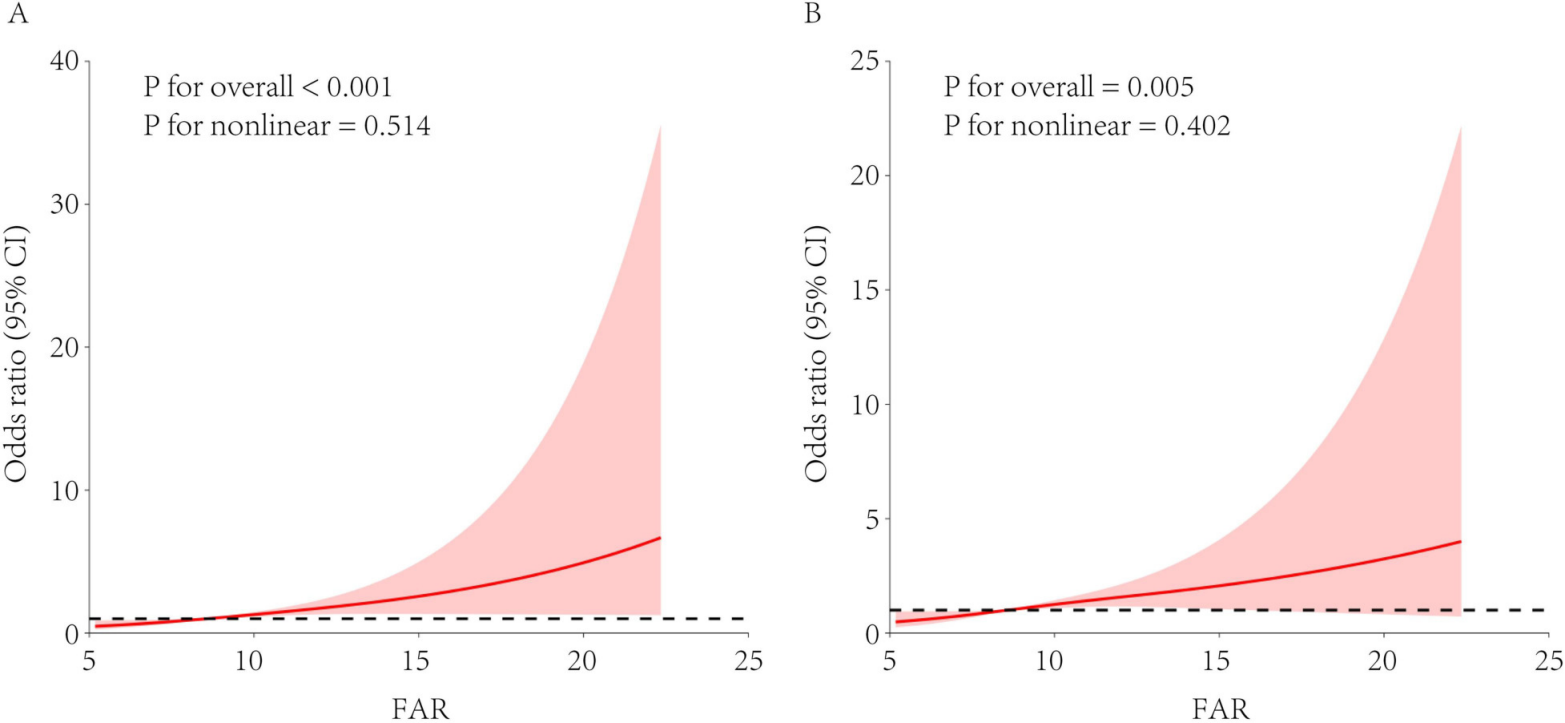
RCS analysis of the association between FAR and arteriosclerosis risk. FAR, fibrinogen-to-albumin ratio; RCS, restricted cubic spline; eGFR, estimated glomerular filtration rate. The restricted cubic spline model used knots at the 10th, 50th, and 90th percentiles. **(A)** Univariate model without adjusting for covariates. **(B)** Multivariate model adjusted for hypertension, diabetes, dyslipidemia, sex, age, and estimated eGFR.

A univariate RCS analysis showed a strong overall association between FAR and arteriosclerosis risk (overall *p* < 0.001), with a predominantly linear trend (*p* for non-linearity = 0.514; Figure 1A). After adjusting for covariates as defined in Model 3, a multivariate RCS analysis confirmed that the independent association between FAR and arteriosclerosis remained significant (overall *p* = 0.005) and the linear trend persisted (*p* for non-linearity = 0.402; Figure 1B).

An interaction analysis (Table 4) revealed no significant interactions between FAR and gender, smoking status, DM, HTN, dyslipidemia, or high BMI in relation to arteriosclerosis (all interaction *p* > 0.05).

**Table 4:** Stratified interaction of FAR and arteriosclerosis.

Variables	Quartile of FAR							*p* for trend	*p* for interaction
	Q1	Q2		Q3		Q4			
	OR (95% CI)	OR (95% CI)	*p-*Value	OR (95% CI)	*p*-Value	OR (95% CI)	*p*-Value		
Gender									
Female	1 (ref)	1.221 (0.345–4.325)	0.757	1.469 (0.394–5.481)	0.567	3.006 (0.655–13.803)	0.157	0.018	0.676
Male	1 (ref)	0.821 (0.366–1.840)	0.632	2.591 (1.054–6.369)	0.038	3.397 (1.256–9.188)	0.016	0.035	
Smoking status									
Non-smoker	1 (ref)	1.135 (0.531–2.427)	0.744	1.847 (0.803–4.250)	0.149	2.699 (1.051–6.929)	0.039	0.020	0.614
Smoker	1 (ref)	0.967 (0.216–4.329)	0.965	4.349 (0.902–20.965)	0.067	4.903 (0.822–29.244)	0.081	0.024	
Diabetes									
No	1 (ref)	1.011 (0.391–2.615)	0.982	2.079 (0.775–5.577)	0.146	2.678 (0.899–7.980)	0.077	0.033	0.676
Yes	1 (ref)	0.783 (0.275–2.231)	0.647	2.118 (0.693–6.474)	0.188	3.335 (0.854–13.017)	0.083	0.036	
Hypertension									
No	1 (ref)	1.712 (0.363–8.078)	0.497	3.038 (0.633–14.582)	0.165	7.809 (1.294–47.112)	0.025	0.018	0.815
Yes	1 (ref)	0.784 (0.370–1.660)	0.525	1.875 (0.815–4.312)	0.139	2.351 (0.917–6.025)	0.075	0.023	
Dyslipidemia									
No	1 (ref)	0.342 (0.101–1.155)	0.084	1.284 (0.332–4.961)	0.717	5.675 (0.923–34.893)	0.061	0.055	0.818
Yes	1 (ref)	1.597 (0.684–3.727)	0.279	2.755 (1.136–6.683)	0.025	2.465 (0.913–6.655)	0.075	0.025	
High BMI									
No	1 (ref)	1.081 (0.403–2.899)	0.877	3.464 (1.152–10.418)	0.027	3.857 (1.100–13.528)	0.035	0.006	0.326
Yes	1 (ref)	1.120 (0.424–2.957)	0.819	1.493 (0.539–4.138)	0.441	2.691 (0.882–8.211)	0.082	0.077	

BMI, body mass index. Adjusted for gender, age, smoking status, hypertension, diabetes, dyslipidemia, high BMI, hypoglycemic agents, antihypertensive agents, antidyslipidemic agents, PLT, ALT, eGFR, and UA.

## 4 Discussion

This study aimed to explore the association between FAR and arterial stiffness. BaPWV is a validated and convenient clinical indicator for assessing arterial stiffness. FAR was independently associated with both baPWV and arteriosclerosis, and these associations remained statistically significant after adjusting for potential confounding factors. A linear dose–response relationship between FAR and arteriosclerosis was further confirmed. A significant difference in the FAR was observed between participants with and without arteriosclerosis. The arteriosclerosis group exhibited a higher mean FAR compared with the non-arteriosclerosis group (10.1 ± 4.3% vs. 8.3 ± 2.8%). Participants with arteriosclerosis were older and more likely to have comorbid cardiovascular risk factors such as diabetes and hypertension. This finding aligns with established clinical patterns, as arterial stiffness increases with age and is often accompanied by metabolic risk factors.

Atherosclerosis is characterized by arterial lumen narrowing and the formation, erosion, and rupture of atherosclerotic plaques (20). These pathological processes directly induce ischemia or thrombosis, thereby contributing to CVD, including peripheral vascular disease, cerebrovascular disease, and coronary heart disease. In severe cases, inadequate collateral circulation may lead to myocardial infarction or cerebral infarction. As a chronic, highly deleterious vascular disorder, atherosclerosis has shown an increasing incidence. Identifying individuals at risk of cardiovascular events remains a key focus of preventive strategies, emphasizing the critical role of early detection and intervention in mitigating disease progression at its initial stages. Arteriosclerosis, characterized by increased arterial stiffness, represents an early marker of atherosclerosis (21). BaPWV is validated and convenient for assessing arterial stiffness, but it is not routinely available in many hospitals and primary-care settings, highlighting the need for a simpler alternative.

Existing evidence supports the relevance of FAR to cardiovascular pathophysiology. For instance, Ozdemir et al. identified FAR as a predictor of exaggerated morning blood pressure surges, a well-recognized CVD risk factor (22). FAR has also been independently associated with coronary artery disease (CAD) severity in patients with ST-segment elevation myocardial infarction (23). Similar associations have been reported in patients with non-ST-segment elevation myocardial infarction (24, 25). Moreover, FAR robustly predicts MACEs in CAD patients undergoing percutaneous coronary intervention (26). Zheng et al. linked FAR to adverse outcomes after lacunar stroke (27). Collectively, these studies position FAR as a risk factor for adverse cardiovascular events.

Notably, the association between FAR and baPWV remains insufficiently explored. In the present study, when FAR and baPWV were analyzed as continuous variables in univariable analyses, FAR was positively correlated with baPWV. Furthermore, when arteriosclerosis was defined as baPWV ≥1,800 cm/s, the prevalence of arteriosclerosis increased progressively across ascending FAR quartiles. These findings indicate that elevated FAR is independently associated with greater baPWV.

The potential mechanisms underlying the association between FAR and arteriosclerosis require further exploration. Arteriosclerosis, an early hallmark of vascular aging, is characterized by reduced vascular wall elasticity and increased stiffness (2). A growing body of evidence supports the concept of “inflammaging,” which links chronic, low-grade inflammation to progressive arterial stiffening (28). Aminuddin et al. reported an association between inflammation and elevated PWV (29). In addition, abnormal hemodynamic changes, including increased blood viscosity and coagulation activation, contribute to arteriosclerotic progression (30). FAR, influenced by both fibrinogen and albumin, may be involved in the interplay of these processes (inflammatory and hemodynamic pathways) that are associated with arteriosclerosis, although the direction of this relationship remains unclear. Fibrinogen, a key inflammatory marker, enhances inflammatory cell adhesion and upregulates proinflammatory cytokine synthesis (31). Elevated fibrinogen levels may increase blood viscosity, potentially inducing shear stress–mediated endothelial damage and coagulation activation (32). Conversely, lower albumin levels correlate with heightened inflammation (11). Inflammation increases vascular permeability and disrupts fluid homeostasis, leading to rapid changes in plasma albumin concentrations (32, 33). Observational data further show an inverse correlation between plasma albumin and C-reactive protein (CRP), a classic inflammatory marker (34). Beyond its role in inflammation, albumin exerts antioxidant effects by scavenging reactive oxygen species (ROS) and limiting oxidative stress (12, 35). Albumin also has anticoagulant properties that help maintain hemostatic balance. Therefore, hypoalbuminemia is associated with a prothrombotic state (36–38). Fibrinogen and albumin may jointly promote systemic inflammation and hemodynamic disturbances, and their alterations may together contribute to the pathophysiological processes underlying arteriosclerosis. As an integrated index, FAR captures the concurrent pattern of elevated fibrinogen and reduced albumin and may therefore be more sensitive than either marker alone in reflecting inflammatory activity and hemodynamic perturbations. In the present study, a higher FAR was associated with an increased risk of arteriosclerosis, suggesting that chronic inflammation and hemodynamic dysregulation may represent key potential pathways linking FAR to arterial stiffening.

Interaction analyses showed that the association between FAR and arteriosclerosis was not significantly modified by gender, smoking status, diabetes, hypertension, dyslipidemia, or high BMI, suggesting that the predictive value of FAR may be broadly applicable in older adults. Even among elderly individuals without overt traditional cardiovascular risk factors, an elevated FAR remained associated with a higher risk of arteriosclerosis, indicating that FAR may serve as a potential early warning marker of vascular aging.

Several limitations in this study should be noted. First, the retrospective cross-sectional design precludes causal inference between FAR and baPWV. Arterial stiffness itself may induce chronic inflammation or disrupt hemodynamic homeostasis, thereby increasing FAR, and reverse causation cannot be excluded. Prospective cohort studies or interventional trials are needed to determine directionality. Second, the generalizability of our findings is limited. The study included older adults aged 65 years or older, and the distribution of FAR and its association with baPWV may differ in adults younger than 65 years who typically have better vascular elasticity and lower baseline inflammation. In addition, only older Chinese participants were included, and differences in genetic background, lifestyle, and disease spectrum may limit extrapolation to other age groups and ethnic populations. Third, the sample size of 389 was adequate for primary correlation and regression analyses, but statistical power for subgroup analyses was limited. Fourth, residual confounding remains possible in this single-center retrospective study. Although we adjusted for routine covariates, we did not account for chronic inflammatory diseases such as rheumatoid arthritis and systemic lupus erythematosus, liver dysfunction, nutritional indicators such as prealbumin and dietary protein intake, inflammatory markers including CRP and IL-6, or relevant medication history, all of which may affect the precision of association estimates. Overall, large-scale, multicenter, multiethnic prospective studies are warranted to further validate the clinical value of FAR as a marker of vascular aging.

## 5 Conclusions

This study provides evidence in older adults that FAR is independently and linearly associated with arterial stiffness, offering a simple and readily available indicator for vascular aging assessment.

## Data Availability

The raw data supporting the conclusions of this article will be made available by the authors, without undue reservation.
