# Supplementary material for: The relationship between fibrinogen-to-albumin ratio and brachial-ankle pulse wave velocity in elderly individuals in China: a cross-sectional study
Source: Front Cardiovasc Med. 2026 Feb 9;13:1737344. doi: 10.3389/fcvm.2026.1737344 (PMC12926422; doi:10.3389/fcvm.2026.1737344)
Supplement: Supplementary file 1 [file Datasheet1.docx]

Supplementary Table 1 Characteristics of patients stratified by the presence or absence of arteriosclerosis.

Supplementary Table 2 Pearson correlation analysis for baPWV.

Supplementary Table 3 Logistic regression of FAR and arteriosclerosis.

Supplementary Table 1 Characteristics of patients stratified by the presence or absence of arteriosclerosis.

|  | All patients  (n=389) | Patients without arteriosclerosis  (n=110) | Patients with arteriosclerosis  (n=279) | *p-*value |
| --- | --- | --- | --- | --- |
| Cardiovascular Risk Factors | | | | |
| Age (year) | 76.5±7.4 | 73.5±6.8 | 77.7±7.3 | < 0.001 |
| Male (n, %) | 247 (63.5%) | 71 (64.5%) | 176 (63.1%) | 0.787 |
| Smokers (n, %) | 101 (26.0%) | 30 (27.3%) | 71 (25.4%) | 0.717 |
| Diabetes (n, %) | 167 (42.9%) | 38 (34.5%) | 129 (46.2%) | 0.036 |
| Hypertension (n, %) | 308 (79.2%) | 76 (69.1%) | 232 (83.2%) | 0.002 |
| Dyslipidemia (n, %) | 261 (67.1%) | 73 (66.4%) | 188 (67.4%) | 0.847 |
| High BMI (n, %) | 186 (47.8%) | 56 (50.9%) | 130 (46.6%) | 0.443 |
| Current Treatments (n, %) | | | | |
| Hypoglycemic agents | 122 (31.4%) | 25 (22.7%) | 97 (34.8%) | 0.021 |
| Antihypertensive agents | 222 (57.1%) | 57 (51.8%) | 165 (59.1%) | 0.189 |
| Antidyslipidemic agents | 63 (16.2%) | 20 (18.2%) | 43 (15.4%) | 0.504 |
| Physical Exam | | | | |
| SBP (mmHg) | 142.3±21.0 | 132.0±17.5 | 146.4±20.9 | < 0.001 |
| DBP (mmHg) | 76.7±11.4 | 75.0±10.1 | 77.4±11.8 | 0.068 |
| MABP (mmHg) | 98.6±12.7 | 94.0±10.9 | 100.4±13.0 | < 0.001 |
| HR (bpm) | 76.7±11.3 | 74.9±11.5 | 77.5±11.1 | 0.045 |
| Laboratory Data | | | | |
| PLT (10^9^/L) | 229.3±81.4 | 212.7±75 | 235.9±83.1 | 0.008 |
| ALT (U/L) | 20.2±11.9 | 21.6±13.4 | 19.7±11.2 | 0.152 |
| AST (U/L) | 20.8±9.4 | 20.6±8.1 | 20.8±9.9 | 0.863 |
| FBG (mmol/l) | 6.9±3.5 | 6.9±4.1 | 6.9±3.2 | 0.987 |
| TG (mmol/l) | 1.4±0.8 | 1.5±0.8 | 1.4±0.8 | 0.758 |
| TC (mmol/l) | 4.4±1.2 | 4.5±1.2 | 4.4±1.2 | 0.237 |
| HDL-C (mmol/l) | 1.1±0.3 | 1.2±0.4 | 1.1±0.3 | 0.172 |
| LDL-C (mmol/l) | 2.9±1.1 | 3.0±1.0 | 2.9±1.1 | 0.475 |
| UA (μmol/l) | 341.1±98.1 | 352.9±99.8 | 336.4±97.3 | 0.134 |
| eGFR (ml/min/1.73m^2^) | 55.9±18.6 | 61.3±16.1 | 53.8±19.2 | < 0.001 |
| Fibrinogen (g/l) | 3.8±1.2 | 3.4±1.0 | 4.0±1.3 | < 0.001 |
| ALB (g/l) | 40.9±4.4 | 42.3±3.9 | 40.4±4.5 | < 0.001 |
| FAR (%) | 9.6±4.0 | 8.3±2.8 | 10.1±4.3 | < 0.001 |
| baPWV (cm/s) | 2117.3±531.6 | 1572.8±156.9 | 2332.0±470.3 | < 0.001 |
| BMI body mass index; SBP systolic blood pressure; DBP diastolic blood pressure; MABP mean arterial blood pressure; HR heart rate; PLT platelet count; ALT alanine aminotransferase; AST aspartate aminotransferase; FBG fasting blood glucose; TG triglyceride; TC total cholesterol; HDL-C high-density lipoprotein-cholesterol; LDL-C low-density lipoprotein-cholesterol; UA uric acid; eGFR estimated glomerular filtration rate; ALB albumin; FAR fibrinogen to albumin ratio; baPWV brachial-ankle pulse wave velocity. | | | | |

Supplementary Table 2 Pearson correlation analysis for baPWV.

| Variables | r | *p-*value |
| --- | --- | --- |
| FAR | 0.219 | < 0.001 |
| Gender (female=0, male=1) | -0.064 | 0.205 |
| Age | 0.312 | < 0.001 |
| Smoking status (nonsmoker=0, smoker=1) | -0.111 | 0.029 |
| SBP | 0.248 | < 0.001 |
| DBP | 0.086 | 0.092 |
| HR | 0.192 | < 0.001 |
| BMI | -0.180 | < 0.001 |
| PLT | 0.184 | < 0.001 |
| ALT | 0.007 | 0.887 |
| FBG | 0.043 | 0.397 |
| TG | 0.001 | 0.986 |
| TC | -0.053 | 0.294 |
| HDL-C | -0.081 | 0.110 |
| LDL-C | -0.041 | 0.420 |
| UA | -0.079 | 0.118 |
| eGFR | -0.220 | < 0.001 |
| FAR fibrinogen to albumin ratio; SBP systolic blood pressure; DBP diastolic blood pressure; HR heart rate; BMI body mass index; PLT platelet count; ALT alanine aminotransferase; FBG fasting blood glucose; TG triglyceride; TC total cholesterol; HDL-C high-density lipoprotein-cholesterol; LDL-C low-density lipoprotein-cholesterol; UA uric acid; eGFR estimated glomerular filtration rate. | | |

Supplementary Table 3 Logistic regression of FAR and arteriosclerosis.

|  | New Model 1 | | New Model 2 | |
| --- | --- | --- | --- | --- |
| FAR | OR (95% CI) | *p-*value | OR (95% CI) | *p-*value |
| Q1 | 1 (ref) |  | 1 (ref) |  |
| Q2 | 0.984 (0.513-1.887) | 0.962 | 1.016 (0.531-1.946) | 0.961 |
| Q3 | 2.001 (1.001-4.002) | 0.049 | 2.011 (1.005-4.024) | 0.048 |
| Q4 | 2.945 (1.325-6.550) | 0.008 | 2.984 (1.340-6.647) | 0.007 |
| *p* for trend |  | 0.002 |  | 0.002 |
| FAR (per 1 %) | 1.141 (1.038-1.254) | 0.006 | 1.143 (1.039-1.259) | 0.006 |
| FAR fibrinogen to albumin ratio; PLT platelet count; ALT alanine aminotransferase; UA uric acid; eGFR estimated glomerular filtration rate.  New Model 1: Adjusted for gender, age, smoking status, hypertension, diabetes, high BMI, dyslipidemia, hypoglycemic agents, antihypertensive agents, antidyslipidemic agents, PLT, ALT, MDRD_CHN_, and UA.  New Model 2: Adjusted for gender, age, smoking status, hypertension, diabetes, high BMI, dyslipidemia, hypoglycemic agents, antihypertensive agents, antidyslipidemic agents, PLT, AST, eGFR, and UA  . | | | | |
